# Supplementary material for: Revised Taxonomy of Rhabdoviruses Infecting Fish and Marine Mammals
Source: Animals (Basel). 2022 May 26;12(11):1363. doi: 10.3390/ani12111363 (PMC9179924; doi:10.3390/ani12111363)
Supplement: Supplementary file 1 [file animals-12-01363-s001.zip › animals-1719250-supplementary/Figure S1A,B Rev.pdf]

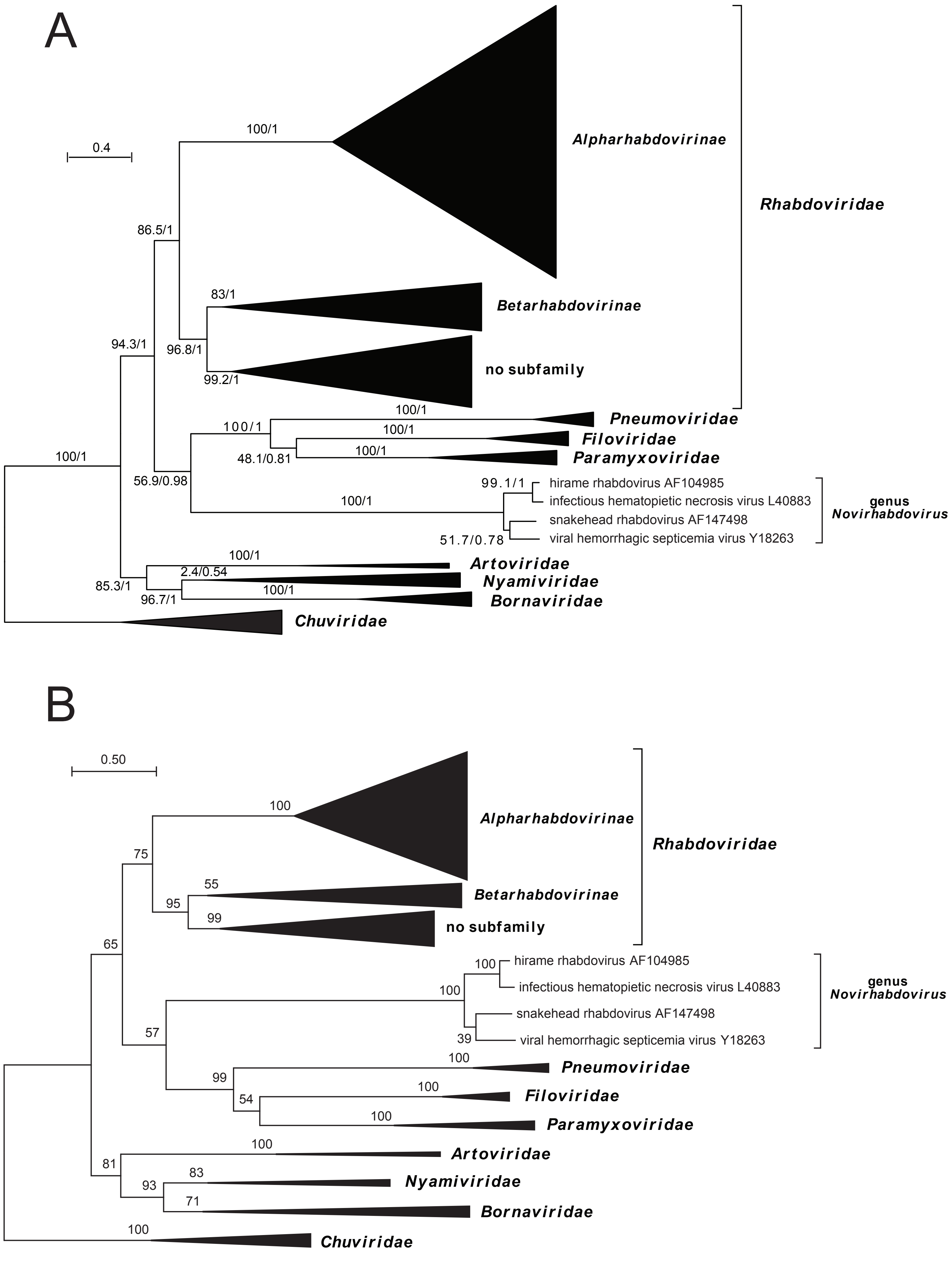
A
0.4
100/1
Alpharhabdovirinae
Rhabdoviridae
86.5/1
83/1
Betarhabdovirinae
94.3/1
96.8/1
no subfamily
99.2/1
100/1
Pneumoviridae
100/1
100/1
Filoviridae
100/1
100/1
Paramyxoviridae
48.1/0.81
56.9/0.98
99.1/1
hirame rhabdovirus AF104985
100/1
infectious hematopietic necrosis virus L40883
genus Novirhabdovirus
snakehead rhabdovirus AF147498
51.7/0.78
viral hemorrhagic septicemia virus Y18263
100/1
Artoviridae
2.4/0.54
Nyamiviridae
85.3/1
100/1
96.7/1
Bornaviridae
Chuviridae
B
0.50
100
Alpharhabdovirinae
75
Rhabdoviridae
55
Betarhabdovirinae
95
99
no subfamily
65
100
hirame rhabdovirus AF104985
100
infectious hematopietic necrosis virus L40883
genus Novirhabdovirus
snakehead rhabdovirus AF147498
57
39
viral hemorrhagic septicemia virus Y18263
100
Pneumoviridae
99
100
Filoviridae
54
100
Paramyxoviridae
100
Artoviridae
81
83
Nyamiviridae
93
71
Bornaviridae
100
Chuviridae

Figure S1. Maximum-likelihood (ML) trees inferred from the same trimmed L protein sequence alignment (916 amino acids) as that used in Figure 2 but using different methods to evaluate branch support. A. ML tree inferred in PhyML using the WAG + Γ amino acid substitution model and the gamma + invariant sites model of site heterogeneity. The best model was selected automatically. The tree with the highest log likelihood (-155217.29) is shown. SH-aLRT support (%) / aBayes support are shown for each branch. B. ML tree inferred in MEGA 7 using the JTT amino acid substitution model. The tree with the highest log likelihood (-164175.08) is shown. Bootstrap support proportion values (500 iterations) are shown for each branch. The trees are rooted on the chuvirus clade and drawn to scale with branch lengths measured in the number of substitutions per site. FigTree was used to plot and annotate the trees.

Any use of trade, firm, or product names is for descriptive purposes only and does not imply endorsement by the U.S. Government.
