# Supplementary material for: Revised Taxonomy of Rhabdoviruses Infecting Fish and Marine Mammals
Source: Animals (Basel). 2022 May 26;12(11):1363. doi: 10.3390/ani12111363 (PMC9179924; doi:10.3390/ani12111363)
Supplement: Supplementary file 1 [file animals-12-01363-s001.zip › animals-1719250-supplementary/Tables S1-S3.pdf]

Table S1. Percentage amino acid sequence divergence (p-distances) estimated from a CLUSTAL W alignment of perhabdovirus, cetarhavirus, siniperhavirus and scophrhavirus L proteins.

**Species**		Virus	1	2	3	4	5	6	7	8	9	10	11	12	13	14	15	16	17	18	19	20	21	22	23	24	25	26	27
Perhabdovirus perca*	1	PRV_Dorson_JX679246																											
	2	PRV_16/065_MN964000	3.8																										
	3	PRV_P8350_MN963998	4.5	3.2																									
Perhabdovirus leman	4	LEMV_18/193_MN963996	14.8	13.4	13.1																								
Perhabdovirus trutta*	5	STRV_R6146_MN963999	19.8	18.8	18.5	18.3																							
	6	STRV_18/203_MN963997	20.0	19.1	18.7	18.4	5.2																						
	7	STRV_CH17_MN510774	20.0	19.0	18.6	18.3	5.0	0.9																					
Perhabdovirus anguilla*	8	EVA_J6B4_KC608038	29.4	28.6	28.2	27.6	28.1	27.7	27.7																				
	9	EVEX_CV1153311_FN557213	29.2	28.4	28.0	27.3	27.5	27.1	27.1	2.1																			
	10	EVEX_C30_JN639009	29.1	28.3	27.9	27.3	27.6	27.2	27.2	1.7	0.6																		
	11	EVEX_DF25/04_KC608037	29.2	28.4	28.0	27.4	27.7	27.2	27.2	2.1	0.9	0.4																	
	12	EVEX_DK3545_KC608034	29.2	28.3	28.0	27.3	27.7	27.2	27.2	1.8	0.9	0.3	0.7																
	13	EVEX_DK3631_KC608035	29.3	28.5	28.1	27.5	27.8	27.3	27.3	2.2	1.1	0.6	1.0	0.8															
	14	EVEX_DK5743_KC608036	29.1	28.3	27.9	27.3	27.6	27.2	27.2	1.8	0.7	0.1	0.5	0.3	0.7														
	15	EVEX_GG129_JN639010	29.3	28.4	28.1	27.4	27.6	27.1	27.1	2.2	1.1	0.6	0.9	0.8	1.1	0.7													
	16	EVEX_GG184_KC608033	29.3	28.4	28.1	27.4	27.6	27.1	27.1	2.2	1.2	0.6	0.9	0.8	1.1	0.8	0.0												
	17	EVEX_JP77_JX827265	70.9	28.3	27.9	27.3	27.6	27.2	27.2	1.7	0.6	0.0	0.4	0.3	0.6	0.1	0.6	0.6											
Cetarhavirus lagalorhynchus	18	DRV_KF958252	44.7	43.8	44.2	44.3	45.0	44.3	44.6	44.1	44.1	44.3	44.2	44.3	44.4	44.3	44.3	44.3	44.3										
Cetarhavirus phocoena	19	HPRV_MN103537	44.8	44.3	44.4	44.5	44.6	44.6	44.8	44.2	44.3	44.2	44.4	44.3	44.3	44.2	44.2	44.2	44.2	31.3									
Siniperhavirus zoarces	20	EPRV_KR612230	48.2	47.7	47.9	47.4	47.8	47.5	47.7	48.5	48.0	48.1	48.1	48.2	48.3	48.1	48.2	48.2	48.1	46.1	47.5								
Siniperhavirus chuatsi	21	HSHV_C1207_KC519324	48.2	47.8	47.7	47.9	47.8	47.6	47.6	48.7	48.4	48.4	48.4	48.4	48.4	48.4	48.4	48.4	48.4	45.8	46.0	28.8							
	22	HSHV_SHVV19_MW291462	48.3	47.8	47.8	47.8	47.8	47.7	47.7	48.7	48.4	48.4	48.4	48.4	48.4	48.4	48.4	48.4	48.4	45.6	45.7	28.9	1.1						
	23	CrERV_MH319839	48.0	47.7	47.5	47.7	47.6	47.5	47.5	48.5	48.2	48.2	48.2	48.2	48.2	48.2	48.2	48.2	48.2	45.8	46.0	29.2	2.4	2.8					
	24	MSRV_MK397811	48.1	47.8	47.5	48.0	47.7	47.5	47.5	48.6	48.3	48.3	48.3	48.3	48.3	48.3	48.3	48.3	48.3	45.8	45.8	29.0	3.0	3.6	3.1				
	25	SCRV_DQ399789	48.4	48.0	47.8	48.3	48.1	47.9	47.9	48.9	48.6	48.6	48.6	48.6	48.6	48.6	48.6	48.6	48.6	46.1	46.1	29.3	2.4	2.9	2.6	2.1			
Scophrhavirus maximus	26	SMRV_HQ003891	53.4	52.8	53.1	53.4	52.9	53.1	53.2	53.8	53.7	53.6	53.6	53.6	53.7	53.6	53.5	53.5	53.6	53.2	53.3	54.0	53.5	53.6	53.4	53.4	53.5		
Scophrhavirus chanodychthys	27	WhRCDRV_MG600013	54.7	54.5	54.2	54.4	54.8	55.4	55.5	55.0	55.1	55.0	55.1	55.1	55.2	55.0	55.1	55.1	55.0	52.8	53.9	55.1	55.3	55.3	55.3	55.1	55.2	46.5	

*Existing species

Table S2. Percentage amino acid sequence divergence (p-distances) estimated from a CLUSTAL W alignment of perhabdovirus, cetarhavirus, siniperhavirus and scophrhavirus N proteins.

Species		Virus	1	2	3	4	5	6	7	8	9	10	11	12	13	14	15	16	17	18	19	20	21	22	23	24	25	26	27	28
Perhabdovirus perca*	1	PRV_Dorson_JX679246																												
	2	PRV_16/065_MN964000	2.7																											
	3	PRV_P8350_MN963998	2.0	2.4																										
Perhabdovirus leman	4	LEMV_18/193_MN963996	15.9	17.4	16.1																									
Perhabdovirus trutta*	5	LTRV_AF434991	24.9	25.9	25.2	27.1																								
	6	STRV_R6146_MN963999	24.9	25.9	25.2	27.4	0.5																							
	7	STRV_18/203_MN963997	25.9	26.9	26.2	27.9	5.9	5.9																						
	8	STRV_CH17_MN510774	26.2	26.9	26.4	28.1	6.1	6.1	1.5																					
Perhabdovirus anguilla*	9	EVA_J6B4_KC608038	37.7	37.2	37.7	38.6	39.9	40.1	40.1	40.3																				
	10	EVEX_CV1153311_FN557213	37.7	37.2	37.7	38.6	39.9	40.1	40.1	40.3	1.5																			
	11	EVEX_C30_JN639009	37.2	36.7	37.2	38.1	39.4	39.6	39.6	39.9	1.0	1.7																		
	12	EVEX_DF25/04_KC608037	37.4	36.9	37.4	38.4	39.6	39.9	39.9	40.1	0.7	1.0	1.0																	
	13	EVEX_DK3545_KC608034	37.2	36.7	37.2	38.1	39.1	39.4	39.4	39.6	1.2	1.7	0.7	1.0																
	14	EVEX_DK3631_KC608035	37.2	36.7	37.2	38.1	39.1	39.4	39.4	39.6	1.0	1.5	0.5	0.7	0.2															
	15	EVEX_DK5743_KC608036	37.2	36.7	37.2	38.1	39.1	39.4	39.4	39.6	1.2	1.7	0.7	1.0	0.0	0.2														
	16	EVEX_GG129_JN639010	37.4	36.9	37.4	38.4	39.4	39.6	39.6	39.9	0.7	1.0	1.0	0.2	0.7	0.5	0.7													
	17	EVEX_GG184_KC608033	37.4	36.9	37.4	38.4	39.4	39.6	39.6	39.9	0.7	1.0	1.0	0.2	0.7	0.5	0.7	0.0												
	18	EVEX_JP77_JX827265	37.2	36.7	37.2	38.1	39.4	39.6	39.6	39.9	0.7	1.5	0.2	0.7	0.5	0.2	0.5	0.7	0.7											
Cetarhavirus lagalorhynchus	19	DRV_KF958252	58.7	58.7	59.4	59.7	58.2	57.9	58.7	58.9	60.6	60.6	60.4	60.4	60.4	60.4	60.4	60.4	60.4	60.4										
Cetarhavirus phocoena	20	HPRV_MN103537	57.7	57.2	57.2	57.2	55.7	56.0	57.0	56.7	56.5	56.2	56.2	56.2	56.2	56.2	56.2	56.2	56.2	56.2	31.8									
Sinieperhavirus zoarces	21	EPRV_KR612230	58.9	59.2	58.4	59.2	59.2	59.4	59.9	59.7	60.9	60.9	60.4	60.6	60.4	60.4	60.4	60.6	60.6	60.4	51.3	53.8								
Siniperhavirus chuatsi	22	HSHV_C1207_KC519324	55.5	55.5	56.2	58.4	56.5	56.2	57.0	56.7	58.2	58.2	58.2	57.7	58.2	58.2	58.2	57.9	57.9	58.2	51.6	53.3	34.2							
	23	HSHV_SHVV19_MW291462	55.5	55.5	56.2	58.4	56.7	56.5	57.2	57.0	58.2	58.2	58.2	57.7	58.2	58.2	58.2	57.9	57.1	58.2	51.8	53.5	34.0	0.7						
	24	CrERV_MH319839	56.2	56.2	56.7	57.7	56.7	56.5	57.2	57.0	57.5	57.5	57.5	57.3	57.5	57.5	57.5	57.2	57.2	57.5	57.2	53.5	33.7	3.7	4.4					
	25	MSRV_MK397811	56.7	56.2	56.7	58.2	57.0	56.7	57.2	57.0	58.2	58.2	57.9	57.9	58.2	58.2	58.2	57.9	57.9	58.2	52.8	53.0	35.7	5.6	6.4	6.8				
	26	SCRV_DQ399789	56.5	56.0	56.7	58.2	57.0	56.7	57.7	57.5	58.7	58.7	58.2	58.4	58.4	58.4	58.4	58.4	58.4	58.4	51.8	53.8	34.7	3.4	4.2	4.2	4.4			
Scophrhavirus maximus	27	SMRV_HQ003891	68.9	68.7	69.2	69.4	71.6	71.6	71.4	71.4	69.9	69.9	69.4	69.7	69.4	69.4	69.4	69.7	69.7	69.4	68.9	68.7	72.9	72.6	72.6	72.1	72.9	72.6		
Scophrhavirus chanodychthys	28	WhRCDRV_MG600013	70.2	69.4	70.2	71.6	71.1	70.9	70.7	70.9	70.4	69.9	69.9	70.2	69.9	69.9	69.9	70.2	70.2	69.9	69.7	70.4	71.9	69.9	69.7	70.2	69.9	69.7	62.6	

*Existing species

Table S3. Percentage amino acid sequence divergence (p-distances) estimated from a CLUSTAL W alignment of perhabdovirus, cetarhavirus, siniperhavirus and scophrhavirus G proteins.

**Species**		Virus	1	2	3	4	5	6	7	8	9	10	11	12	13	14	15	16	17	18	19	20	21	22	23	24	25	26	27	28
Perhabdovirus perca*	1	**PRV_Dorson_JX679246**																												
	2	PRV_16/065_MN964000	3.3																											
	3	PRV_P8350_MN963998	1.8	3.1																										
Perhabdovirus leman	4	LEMV_18/193_MN963996	16.9	18.0	16.7																									
Perhabdovirus trutta*	5	**LTRV_903/87_AF434991**	30.4	31.0	30.8	32.4																								
	6	STRV_R6146_MN963999	30.2	31.0	30.6	32.0	4.3																							
	7	STRV_18/203_MN963997	32.0	32.2	32.0	31.8	10.4	11.6																						
	8	STRV_CH17_MN510774	32.0	32.7	32.0	31.8	9.6	11.0	2.7																					
Perhabdovirus anguilla*	9	EVA_J6B4_KC608038	46.1	46.3	46.7	47.8	47.6	48.0	46.9	47.3																				
	10	**EVEX_CV1153311_FN557213**	45.9	46.7	46.5	48.0	47.3	47.6	47.6	47.6	3.3																			
	11	EVEX_C30_JN639009	45.9	46.7	46.5	48.0	47.6	47.8	47.8	47.8	3.3	0.4																		
	12	EVEX_DF25/04_KC608037	46.1	46.7	46.7	48.0	47.3	47.6	47.3	47.3	3.5	1.0	1.0																	
	13	EVEX_DK3545_KC608034	45.5	46.3	46.1	47.6	47.1	47.3	47.1	47.1	3.5	0.8	0.8	1.2																
	14	**EVEX_DK3631_KC608035**	45.5	46.3	46.1	47.8	47.6	47.8	47.8	47.8	3.9	2.2	2.2	2.7	1.8															
	15	EVEX_DK5743_KC608036	46.5	47.3	47.1	48.6	48.2	48.4	48.4	48.4	4.1	1.2	0.8	1.8	1.6	3.1														
	16	EVEX_GG129_JN639010	46.1	46.9	46.7	48.0	47.6	47.8	47.8	47.8	3.7	0.8	0.8	1.4	1.2	2.7	1.6													
	17	EVEX_GG184_KC608033	46.1	46.9	46.7	48.0	47.6	47.8	47.8	47.8	3.5	0.6	0.6	1.2	1.0	2.4	1.4	0.2												
	18	EVEX_JP77_JX827265	45.9	46.7	46.5	48.0	47.3	47.6	47.3	47.3	3.3	0.2	0.4	1.0	0.8	2.2	1.2	0.8	0.6											
Cetarhavirus lagalorhynchus	19	DRV_KF958252	67.3	67.1	67.3	66.7	68.8	69.2	67.1	67.3	68.2	68.6	68.6	68.4	68.4	68.4	69.0	69.0	68.8	68.6										
Cetarhavirus phocoena	20	HPRV_MN103537	66.5	66.3	66.7	66.3	68.4	68.4	68.0	68.0	66.9	67.3	67.3	67.1	67.3	67.6	67.8	67.6	67.6	67.3	56.1									
Siniperhavirus zoarces	21	EPRV_KR612230	72.9	72.7	73.1	73.3	75.5	74.9	75.3	74.9	71.4	71.4	71.4	71.2	71.4	71.2	71.6	71.8	71.6	71.2	71.2	73.7								
Siniperhavirus chuatsi	22	HSHV_C1207_KC519324	73.1	73.1	73.3	72.7	73.1	72.7	73.5	73.7	72.7	72.4	72.4	72.2	72.2	72.0	72.7	72.7	72.4	72.4	69.4	70.4	50.4							
	23	HSHV_SHVV19_MW291462	73.1	73.1	73.3	73.1	73.1	72.7	73.9	74.1	73.3	73.1	73.1	72.9	72.9	72.7	73.7	73.3	73.1	73.1	69.8	71.2	50.0	3.9						
	24	CrERV_MH319839	72.7	72.7	72.9	72.4	73.7	73.5	74.3	74.5	72.9	72.7	72.7	72.7	72.4	72.2	72.9	72.9	72.7	72.7	70.0	71.2	50.0	8.6	10.6					
	25	MSRV_MK397811	72.9	72.7	73.1	72.9	74.1	73.9	74.9	75.3	73.3	73.3	73.3	73.1	73.1	73.9	73.5	73.5	73.3	73.3	69.6	71.6	51.8	8.6	10.6	8.6				
	26	SCRV_DQ399789	72.2	72.2	72.4	72.4	73.9	73.7	74.9	75.1	73.3	73.1	73.1	72.9	72.9	72.7	73.3	73.3	73.1	73.1	69.2	72.0	51.2	6.3	8.4	6.7	3.1			
Scophrhavirus maximus	27	SMRV_HQ003891	78.0	78.2	78.4	77.6	78.8	78.6	77.8	77.8	76.1	76.3	76.3	76.1	76.3	76.9	76.5	76.3	76.3	76.3	78.2	77.8	79.8	80.0	80.8	78.8	81.0	80.6		
Scophrhavirus chanodychthys	28	WhRCDRV_MG600013	74.4	73.9	74.7	75.1	75.1	75.5	75.5	75.3	74.9	74.5	74.5	74.3	74.3	74.3	75.1	74.7	74.7	74.5	76.7	77.8	79.0	79.0	78.8	78.6	78.8	79.4	71.0	

*Existing species
